# Supplementary material for: Research the Thermal Decomposition Processes of Copolymers Based on Polypropyleneglycolfumaratephthalate with Acrylic Acid
Source: Polymers (Basel). 2023 Mar 30;15(7):1725. doi: 10.3390/polym15071725 (PMC10096502; doi:10.3390/polym15071725)
Supplement: Supplementary file 1 [file polymers-15-01725-s001.zip › Figure S1.pdf]

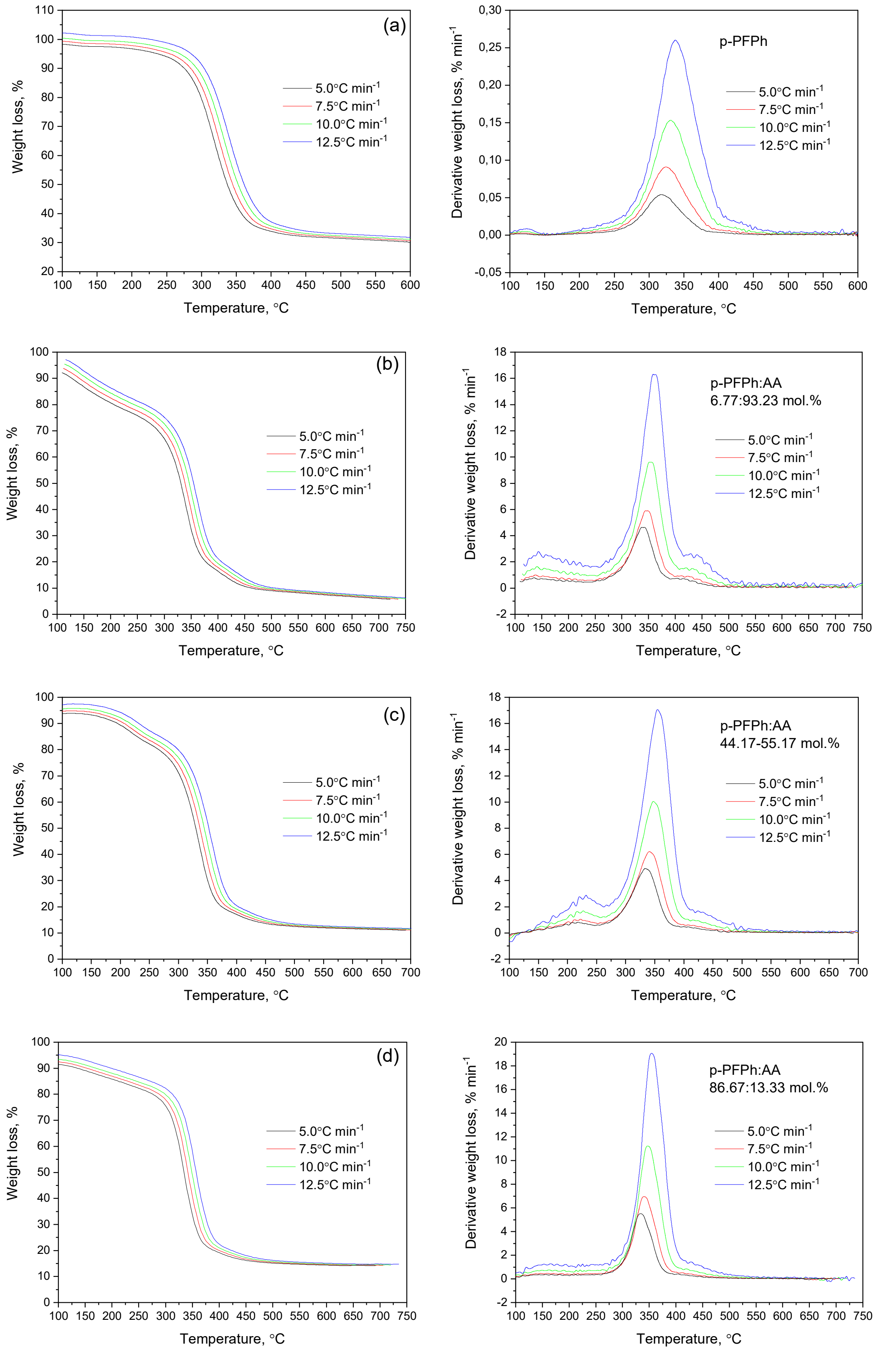

Figure S1 TG and dTG curves of p-PFP and p-PFP:AA copolymers at initial ratios M_1:M_2, mol.%:
(a)–p-PFP; (b)–6.77:93.23; (c)–44.17:55.17 and (d)–86.67:13.33 (in nitrogen environment)
