# Supplementary material for: Research the Thermal Decomposition Processes of Copolymers Based on Polypropyleneglycolfumaratephthalate with Acrylic Acid
Source: Polymers (Basel). 2023 Mar 30;15(7):1725. doi: 10.3390/polym15071725 (PMC10096502; doi:10.3390/polym15071725)
Supplement: Supplementary file 1 [file polymers-15-01725-s001.zip › Figure S2.pdf]

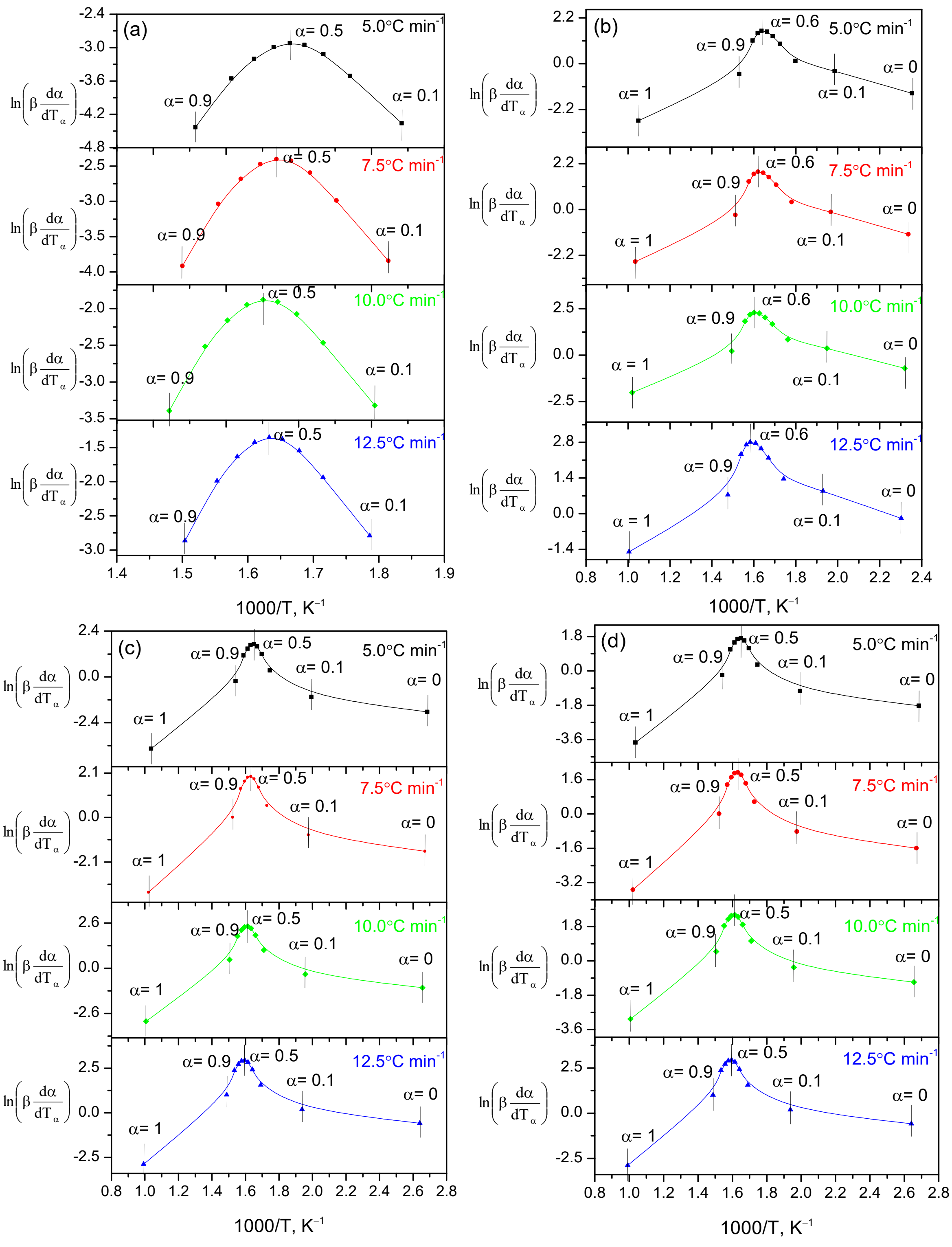

Figure S2 Dependence diagrams $\ln\left[\beta \frac{d\alpha}{dT_\alpha}\right]$ on 1/T determined using Friedman method (a)–p-PFP; (b)–6.77:93.23; (c)–44.17:55.17 and (d)–86.67:13.33
